# Accuracy investigations for volumetric head‐motion navigators with and without EPI at 7 T

**DOI:** 10.1002/mrm.29296

**Published:** 2022-05-16

**Authors:** Mads Andersen, Malte Laustsen, Vincent Boer

**Affiliations:** ^1^ Philips Healthcare Copenhagen Denmark; ^2^ Lund University Bioimaging Center, Lund University Lund Sweden; ^3^ Center for Magnetic Resonance, Department of Health Technology Technical University of Denmark Lyngby Denmark; ^4^ Danish Research Center for Magnetic Resonance, Centre for Functional and Diagnostic Imaging and Research Copenhagen University Hospital – Amager and Hvidovre Copenhagen Denmark

**Keywords:** 7 T, brain, EPI distortions, motion correction, multi‐echo, navigators

## Abstract

**Purpose:**

Accuracy investigation of volumetric navigators for motion correction, with emphasis on geometric EPI distortions at ultrahigh field.

**Methods:**

High‐resolution Dixon images were collected in different head positions and reconstructed to water, fat, T_2_*, and B_0_ maps. Resolution reduction was performed, and the T_2_* and B_0_ maps were used to apply effects of TE and EPI distortions to simulate various volumetric water and fat navigators. Registrations of the simulated navigators were compared with registrations of the original high‐resolution images.

**Results:**

Increased accuracy was observed with increased spatial resolution for non‐EPI navigators. When using EPI, the distortions had a negative effect on registration accuracy, which was most noticeable for high‐resolution navigators. Parallel imaging helped to alleviate those caveats to a certain extent, and 5‐fold acceleration gave close to similar accuracy to non‐EPI in most cases. Shortening the TE by partial Fourier sampling was shown to be mostly beneficial, except for water navigators with long readout durations. The EPI blip direction had an influence on navigator accuracy, and positive blip gradient polarities (yielding mostly image stretching frontally) typically gave the best accuracy for water navigators, whereas no clear recommendation could be made for fat navigators. Generally, fat EPI navigators had lower accuracy than water EPI navigators with otherwise similar parameters.

**Conclusions:**

Echo planar imaging has been widely used for MRI navigators, but the induced distortions reduce navigator accuracy at ultrahigh field. This study can help protocol optimization and guide the complex tradeoff between resolution and EPI acceleration in navigator parameter setup.

## 1 INTRODUCTION

Subject movements can lead to severe artifacts in MR brain imaging. This leads to many failed scans in clinical practice, resulting in a need to repeat scans, and the use of anesthesia in children.^1^, ^2^, ^3^ For MRI at 7 T, spatial resolution is often higher, but to obtain true increased spatial resolution, even small movements cannot be tolerated.

A solution to compensate for motion is the interleaving of very fast, but low‐resolution volumetric scans into the long high‐resolution (host) scan. Realignments of these low‐resolution navigator scans enable rigid‐body motion correction of the host scan. This can be done either prospectively, by updating the scan orientation and position for the host scan during scanning, or retrospectively, by regridding the k‐space segments of the host scan.

Both the prospective and retrospective approaches require that the fast, low‐resolution navigators provide accurate head motion estimates. Several navigator sequences have been proposed in literature, of which the most widely used 3D sequences are volumetric EPI “vNavs”^4^ and non‐EPI but fat‐selective gradient echo “FatNavs.”^5^ The combination of 3D EPI and fat selectivity has also been proposed.^6^

The development of suitable accurate navigator scans is a complex task, as there are several choices that need to be made on parameters such as resolution, use of EPI, partial Fourier and TE, tip angle, fat (or water) selective excitation, and use of parallel imaging. These parameters should be optimized to only minimally affect the host sequence duration, contrast, and SNR, while at the same time get the most accurate motion estimates. Gallichan and Marques^7^ thoroughly investigated the effect of resolution and parallel‐imaging acceleration on navigator accuracy for 3D gradient‐echo water and fat navigators. In this paper we extend the analysis to include the use of EPI on navigator scans, as this aids to further accelerate acquisition, and thus allow for fast high‐resolution navigators. These could potentially reach more accurate motion estimates or enable navigators in smaller time gaps of the host scan. However, as the acquisition bandwidth in the EPI blip direction is low, B_0_ inhomogeneities cause severe local geometric distortions in the blip direction, especially near air‐filled cavities. The distortions change with head orientation and position, and therefore affect navigator registration accuracy for several reasons.^8^ First, the distortions are only significantly present in the blip direction; therefore, the distortions will look different depending on the head orientation relative to the blip direction. Second, the B_0_ inhomogeneities are caused by the tissue susceptibility distribution and can be approximated as a convolution of the tissue susceptibility with a dipole kernel pointing in the direction of the B_0_ field.^9^, ^10^ Thus, the B_0_ inhomogeneities will change with a nodding (“pitch”) or “roll” movement, as the tissue distribution is then rotated relative to the direction of the B0 field. Third, static B_0_ shimming is typically applied to reduce the B_0_ inhomogeneities inside the head, making the background field, which the head is moving through, inhomogeneous. Therefore, rotations around the B_0_ field direction (“yaw” or “shaking” movements) as well as translations can lead to B_0_ changes in the head frame of reference. The magnetic susceptibility effects scale with field strength and EPI navigator accuracy are therefore especially relevant to evaluate at 7 T compared with lower field strengths. Signal dropout due to T_2_* dephasing is another severe artifact with EPI due to the long required TEs. This effect is also increased at ultrahigh field, as T_2_* decreases with field strength. The variations of B_0_ with head orientation/position make the T_2_*, and thereby dropout, change with head position/orientation, potentially also affecting navigator accuracy.

One can, to some extent, experimentally compare the accuracy of motion correction using different techniques. Interleaving different navigators or comparing to optical tracking for specific navigators^11^ with different head positions is a viable approach, but the number of navigators one can compare this way are limited to a few, due to subject compliance. Asking the subject to remain still and instead changing offsets and angulations of the navigators by known amounts is a well‐controlled alternative,^12^ but has important limitations when investigating the effects of EPI distortions, due to the B_0_ and T_2_* changes with head position described previously. Controlled motion using a phantom is a third approach, but as the navigator registration accuracy depends on the image content, the phantoms are required to have MR properties and shapes very similar to human heads, which is not trivial to produce. Therefore, to investigate the accuracy of different types of navigators, within the large space of possible navigator settings, we performed a simulation study. For this we collected high‐resolution MR images in different head positions. By using a 10‐echo Dixon acquisition, we could reconstruct water, fat, T_2_*, and B_0_ maps for each of the head positions. This information was used to simulate, in total, 290 different navigators, of which the motion registration results were compared with the original, high‐resolution “gold standard.” We simulated water and fat navigators of different resolutions with/without EPI for both blip directions (+/−), the effect of different SENSE factors on distortions/dropout, with/without partial Fourier to shorten TEs, and with/without cropping of navigator FOV in the feet–head direction to remove neck and mouth regions with nonrigid motion and poor image quality. Finally, to exemplify what such navigator accuracy differences can do to image quality, we performed retrospective motion‐corrected reconstructions of an in vivo data set from a 3D T_1_‐weighted MPRAGE sequence with two different navigators interleaved. Parts of this work have previously been presented in abstract form.^13^

## 2 METHODS

The study was approved by the local ethics committee (H‐17033372), the Danish Medicines Agency (case number 2017070099), and monitored by the local good clinical practice unit. All participants provided written informed consent before examination. Imaging was performed with the national Danish whole‐body 7T MRI system (Achieva; Philips Healthcare, Best, The Netherlands) located at Hvidovre Hospital, Copenhagen, Denmark, using a two‐channel transmit volume head coil in quadrature mode, with a 32‐channel receiver array (Nova Medical, Wilmington, MA, USA).

### 2.1 Movement experiments with multi‐echo Dixon acquisitions

Experiments were performed on 5 healthy volunteers (2 females). Two Dixon time series with 20 volumes each were acquired per subject. Subjects were asked to move their head to a new position between each volume acquisition, but to remain still during acquisition. For one of the time series, the subjects were asked to perform shaking movements (head rotation around the superior–inferior axis), and for the other series the subjects were asked to perform nodding movements (head rotation around the left–right axis).

The Dixon imaging sequence was an axial slab‐selective, 3D, RF‐spoiled, multi‐echo gradient‐echo sequence with 10 echoes at alternating readout gradient polarities and TE = 1.33 + n*1.3 ms, *n* = 0…9. The isotropic voxel size was 2 mm, FOV was 256 × 256 mm^2^ in plane and 230 mm in the feet–head direction, where an oversampling factor of 1.4 was applied. Additional parameters were as follows: tip angle = 10º, TR = 14.6 ms, bandwidth (BW)/pixel = 1554 Hz, readout direction right–left, SENSE factors of 2.5 × 2.5, linear phase‐encoding order, and an elliptical k‐space shutter was applied, resulting in a scan duration of 38.5 s per volume. Second‐order B_0_ shimming was performed based on a separate whole‐brain B_0_ field map acquired in the starting position before the first experiment (two interleaved 3D gradient‐echo acquisitions, isotropic voxel size of 3.75 mm, TE1/TE2/TR = 2/3/10 ms).

### 2.2 Navigator simulation

**FIGURE 1:**
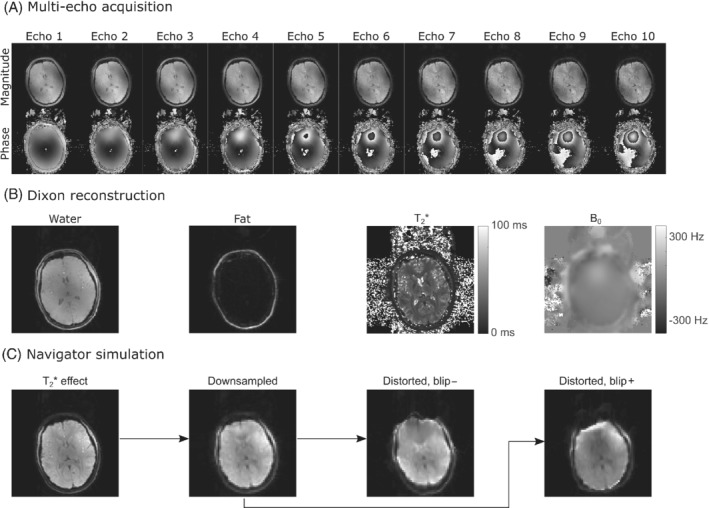
Navigator simulation pipeline. For each head position, a 10‐echo gradient‐echo volume was acquired (A), from which water, fat, T_2_*, and B_0_ maps were calculated using a Dixon reconstruction algorithm (B). The water and fat images were then artificially affected by T_2_*, down‐sampled, and warped depending on the B_0_ field to simulate different navigators (C).

From the multi‐echo volumes, water, fat, B_0_, and T_2_* maps were calculated by the scanner software using the “mDixon quant” option (Figure 1A,B). In the navigator simulation pipeline (Figure 1C), first the effect of the TE was simulated by multiplying the water and fat maps voxel‐wise by $e^{-\frac{\mathrm{TE}}{T_{2}*}-i\;2\pi B_{0}\mathrm{TE}}$, using the corresponding values from the B_0_ and T_2_* maps. Different resolutions were then simulated by cropping the matrix in k‐space, and applying an apodization filter with the same specifications as in the scanner reconstruction, to reduce ringing. To simulate geometric distortions in the anterior–posterior direction, first a voxel displacement map was calculated by multiplying the EPI readout time with the B_0_ map. Signal unwarping algorithms are widely available, although here a forward warping algorithm is required. Therefore, we implemented a custom 1D warping algorithm as follows. For every voxel, convolution with a Dirac delta function was performed, then a Fourier transform along the anterior–posterior direction, a multiplication by a linear phase ramp with a slope proportional to the intensity in the voxel displacement map, followed by an inverse Fourier transform. Summing the result from all pixels yielded the distorted image. Our *MATLAB* (MathWorks, Natick, MA, USA) scripts for the navigator simulation are available online at https://resources.drcmr.dk/moco/.

Navigators of different resolutions were simulated without EPI (no distortions and TE = 0 ms) and with single‐shot EPI readout durations corresponding to triangular readout gradients for a gradient with 40 mT/m gradient strength and 200 T/m/s slew rate. Table 1 lists the resolutions, BW/pixel, and TEs for the EPI simulations. Higher parallel‐imaging factors in the EPI direction can be used to reduce distortions; therefore, bandwidths corresponding to parallel‐imaging accelerations of 1 (= no parallel imaging), 3, and 5 were simulated. Both positive and negative blip gradient polarities were simulated, to investigate the effect of the direction of the geometric distortions. Echo times in the center of the readout were simulated, as well as TEs corresponding to a partial Fourier fraction of 0.75. Fat navigators with TE > 9 ms were not considered, as the short T_2_* of fat would make such navigators too low SNR for practical use. Finally, the effect of cropping the FOV to 160 mm in the feet–head dimension was examined to exclude nonrigid body motion from the neck and mouth.

**TABLE 1:** Bandwidths (BW) and TEs for the simulated EPI navigators. For each resolution, the BW/pixel and TE for each SENSE factor was estimated, assuming triangular readout gradients with 40 mT/m strength and 200 mT/m slew rate. The corresponding image distortion in mm shift per kHz off resonance is also listed. Echo times in the center of the readout, and with PF with acquisition fraction of 0.75, were simulated.

Voxel size (mm)	SENSE factor	BW/ pixel (Hz)	Distortions (mm/kHz)	TE without PF (ms)	TE with PF (ms)
2	1	13.7	146	39	20
2	3	41.0	49	14	8
2	5	68.4	29	9	6
2.7	1	22.3	121	24	13
2.7	3	66.8	40	9	6
2.7	5	111.3	24	6	4
4	1	43.2	93	14	8
4	3	129.6	31	6	4
4	5	216.0	19	4	3
5.3	1	69.8	76	9	6
5.3	3	209.6	25	4	3
5.3	5	349.3	15	3	3
8	1	133.8	60	6	4
8	3	401.4	20	3	3
8	5	669.3	12	3	2
10.7	1	196.7	54	5	3
10.7	3	590.3	18	3	2
10.7	5	984.2	11	3	2

Note that although the SNR improvement with larger voxel size was simulated, there are more SNR effects present in practice that were not simulated. These include the SNR loss with TE, which was not simulated as we multiply both the signal and noise by exp(−TE/T_2_*), the SNR dependence on readout bandwidth, the SNR dependence on SENSE factor, and SNR dependence on partial Fourier factor.

### 2.3 Evaluation of navigator accuracy

As a gold‐standard navigator, the original water images from the Dixon acquisitions with 2‐mm resolution were used after brain masking using the brain segmentation feature of *SPM12* (https://www.fil.ion.ucl.ac.uk/spm/) on each individual volume in the series. The brain masking was performed to ensure the gold standard closely reflected the movement of brain, and to exclude potential confounding effects such as nonrigid motion in the neck and mouth regions, or relative motion between skin and skull at the contact points between head and cushion.

All simulated navigator series (of 20 volumes each) were registered to the first volume in the series. Image registration was performed using the same realignment routine that is present on the scanner and used for prospective motion correction.^14^ This is a rigid body registration based on a mean squared error cost function with Gauss‐Newton optimization, and the output motion parameters are defined with center of the volume as the point of reference. As the focus of this work lies on the more fundamental accuracy limitations of the navigators, rather than the accuracy with a fast, time‐limited realignment, the realignment algorithm was stopped at 500 iterations, or if the change in realignment parameters from iteration to iteration was less than 0.005 mm or degrees.

The estimated motion parameters from the navigators and gold standard were subtracted, resulting in navigator errors in all six motion parameters. To convert this to one parameter describing the size of the motion, the motion score parameter by Tisdall et al^4^ was used, which is defined as

$$\begin{array}{cc} & \text{motion score}=64\;\mathrm{mm}\;\sqrt{{\left[1‐cos(|\theta |)\right]}^{2}+\sin^{2}(|\theta |)\;}\\ & \;+\sqrt{\Delta x^{2}+\Delta y^{2}+\Delta z^{2}} \end{array}$$

where Δx,Δy,andΔz are the translation parameters, and θ is the rotation angle in the axis‐angle representation, which can be computed from the three rotation parameters as follows:

$$\begin{array}{cc} & \theta =\arccos\left\{\frac{1}{2}\left(-1+\cos\left(\theta_{x}\right)\cos\left(\theta_{y}\right)+\cos\left(\theta_{x}\right)\cos\left(\theta_{z}\right)\right.\right.\\ & \left.\left.\;+\cos\left(\theta_{y}\right)\cos\left(\theta_{z}\right)+\sin\left(\theta_{x}\right)\sin(\theta_{y}\right)\sin\left(\theta_{z}\right)\right)\}. \end{array}$$

If a sphere of 64‐mm radius (approximating the size of a human brain) is moved, the motion score will be the maximum displacement experienced across all points on the sphere surface.

The motion score of the navigator errors and the gold‐standard motion parameters were computed, allowing plots of navigator errors versus size of motion for each navigator. Linear least‐squares fits were then estimated separately for nodding and shaking experiments, after rejecting all data points with a gold‐standard rotation parameter > 10º, as outliers were observed for large rotations and such large rotations are rare and difficult to correct. The fit value at 10‐mm motion size was then used for comparing the accuracy of the many different simulated navigators.

### 2.4 Retrospective motion correction of MPRAGE

Possible effects of navigator accuracy on image quality were demonstrated in a single volunteer by retrospectively correcting a T_1_‐weighted 3D‐MPRAGE acquisition with two different navigators. The nonselective 3D‐MPRAGE scan had an isotropic resolution = 0.8 mm, FOV = 212 × 244 × 190 mm^3^, TR/TE/TI = 6/2.6/1200 ms, excitation tip angle = 7º, time between inversions = 3669 ms, turbo factor = 305 (single shot), readout direction = feet–head, no partial Fourier or parallel imaging, and scan duration = 14 min 33 s. Two consecutive navigators were inserted in the T_1_ relaxation delay following the readout train, using the Philips interleaved scanning framework.^15^ A gradient‐echo fat‐selective navigator with 4‐mm isotropic resolution, FOV = 240 × 240 × 240 mm^3^, TR/TE = 3.3/1.5 ms, tip angle = 2º, binomial 1331 excitation, partial Fourier factors of 0.75 × 0.75, and SENSE factors of 3 × 3, yielding a volume scan time of 788 ms. The other navigator was a 3D‐EPI navigator with 8‐mm isotropic resolution, FOV = 256 × 256 × 256 mm^3^, EPI factor = 31 (single shot) with negative blip gradient polarity, phase‐encoding bandwidth = 88.4 Hz/Px, TR/TE = 11/4 ms, tip angle = 2º, partial Fourier factors of 0.75 × 0.75, and no parallel imaging, yielding a volume scan time of 316 ms. The navigators were chosen for demonstration, as the simulations showed clearly different accuracy for the two. Similar non‐EPI 4‐mm fat navigators have been used in other studies at 7 T,^7^, ^11^, ^16^ and the 8‐mm 3D‐EPI water navigator is very similar to the vNAVs^4^ that have been widely used at 3 T (although potentially with different phase‐encoding bandwidth and blip gradient polarity, as they are not clearly reported). The retrospective motion correction of the MPRAGE was performed using the RetroMoCo‐Box,^5^ with one motion update for each MPRAGE readout train based on either of the navigator motion traces as well as a reconstruction without motion correction. A second scan without instructed motion was performed for comparison.

## 3 RESULTS

**FIGURE 2:**
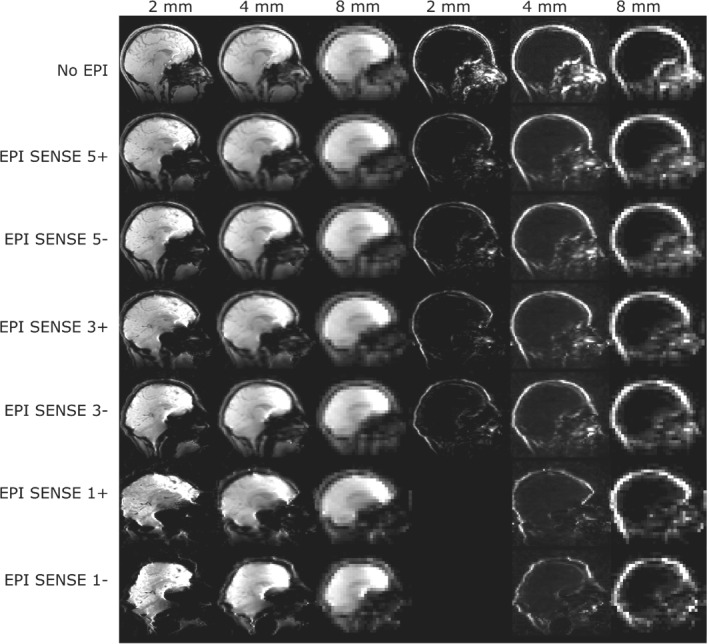
Examples of simulated navigators using partial Fourier (PF). Water navigators are shown on the left, fat navigators on the right. Fat navigators were not simulated for TEs larger than 9 ms, because of the short ${T_{2}}^{*}$ of fat. Navigators were simulated with different resolutions, and both with and without the use of EPI. For EPI, different acceleration (SENSE) factors were investigated to reduce distortions, which occur especially with long readouts, as seen from the higher‐resolution scans with low SENSE factors. Both positive and negative blip gradient polarities were investigated, as this inverts the direction of the distortion (indicated with + and −).

Examples of simulated navigators with partial Fourier are shown in Figure 2. With EPI, the geometric distortions, signal dropout, and T_2_* contrast clearly increase as SENSE factor is decreased. The effects are most striking for the 2‐mm water navigator, which requires long readouts and TEs to cover the large k‐space matrix. A simulated and measured EPI fat navigator with the same parameters is shown in Supporting Information Figure S1. Distortions look similar, but the acquired fat navigator is more noisy, which is expected as SNR effects were not fully simulated.

**FIGURE 3:**
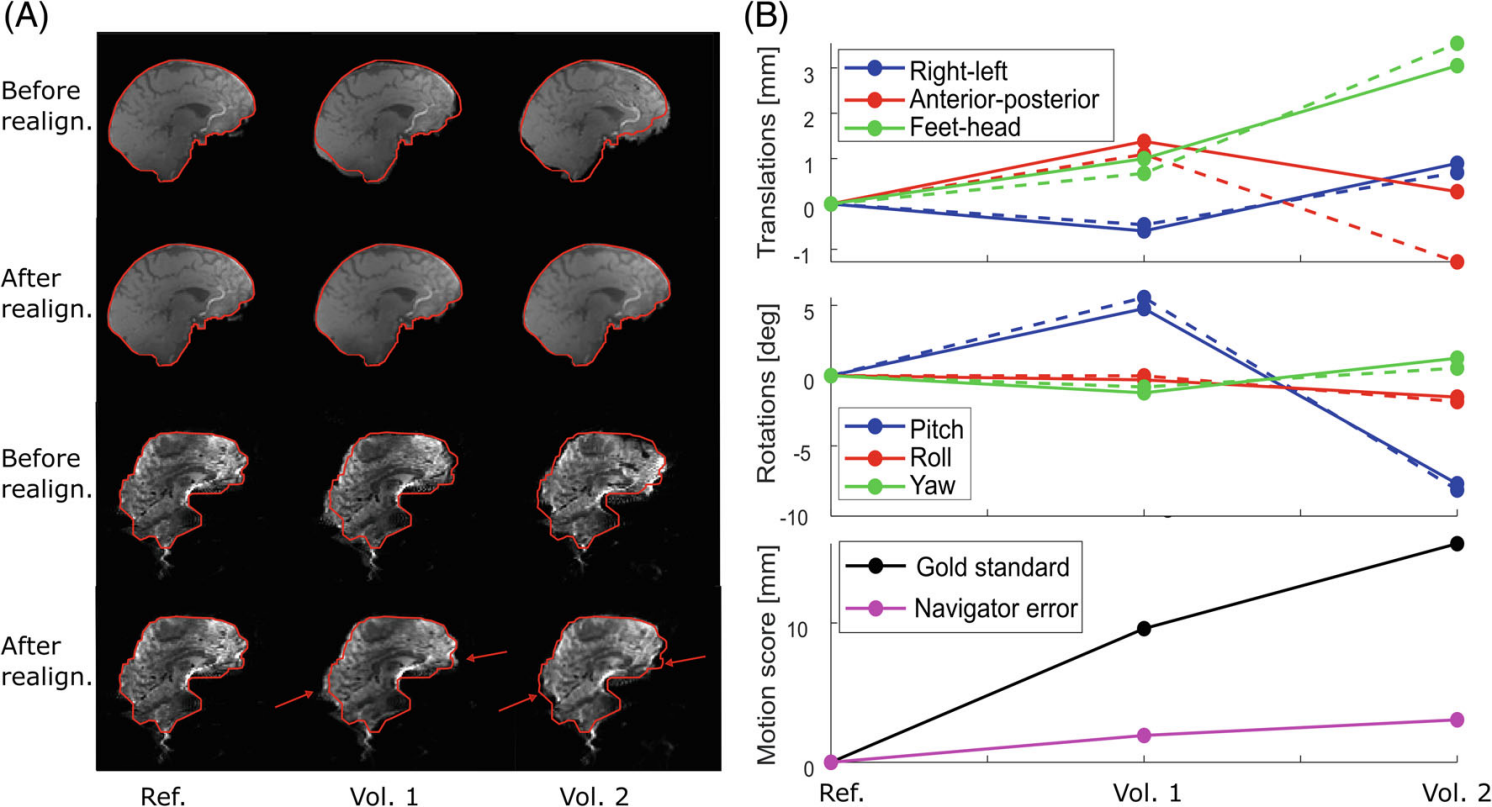
A, Example of realignments for a subject with nodding motion. The gold‐standard volumes are shown on top, and the two lowest panels show the 2‐mm EPI‐SENSE 1‐ water navigator without PF. The red contour is from the reference (ref) volumes to which the others are realigned. B, The corresponding realignment parameters (solid lines indicate the gold standard; dashed lines for the distorted navigator). For the distorted navigator, the geometric distortions change with head position, which challenges the realignment.

The challenge with realignments of geometrically distorted images is illustrated in Figure 3, which shows the results from the realignment of two different volumes to a reference for both the 2‐mm “gold standard” navigator and a distorted 2‐mm EPI navigator. For the distorted navigator, the shape of the brain is changed after movement (see arrows). All six realignment parameters are affected, but the most difference is seen in the anterior–posterior displacement.

**FIGURE 4:**
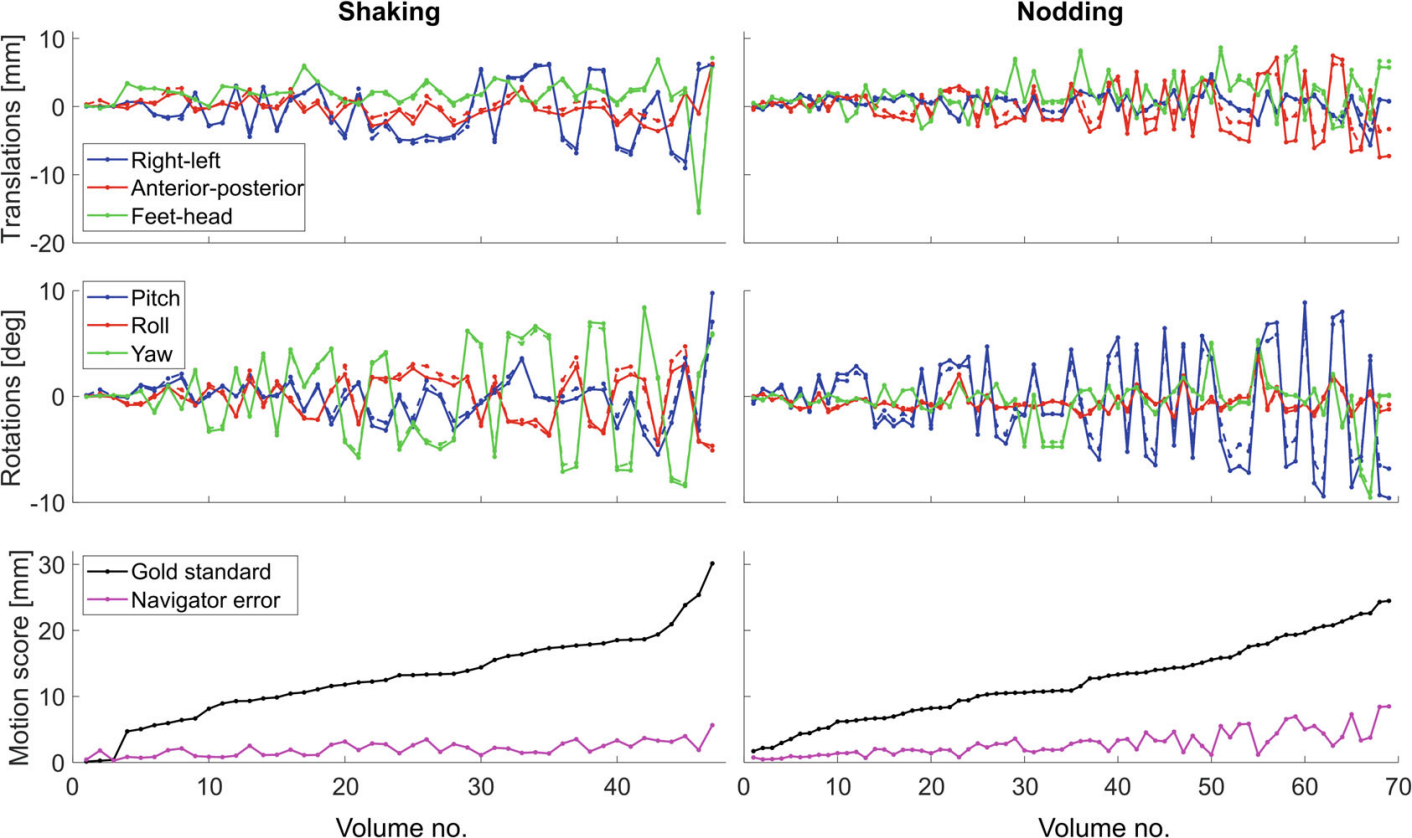
Realignment parameters for gold standard (solid lines) and an 8‐mm EPI SENSE 1‐ water navigator with PF (dashed lines). The bottom panel shows the motion score of the gold‐standard parameters, and the motion score of the difference between the gold standard and navigator realignment parameters (navigator error). Parameters from all volumes used for further analysis are shown, and volumes are ordered by increasing gold‐standard motion score.

**FIGURE 5:**
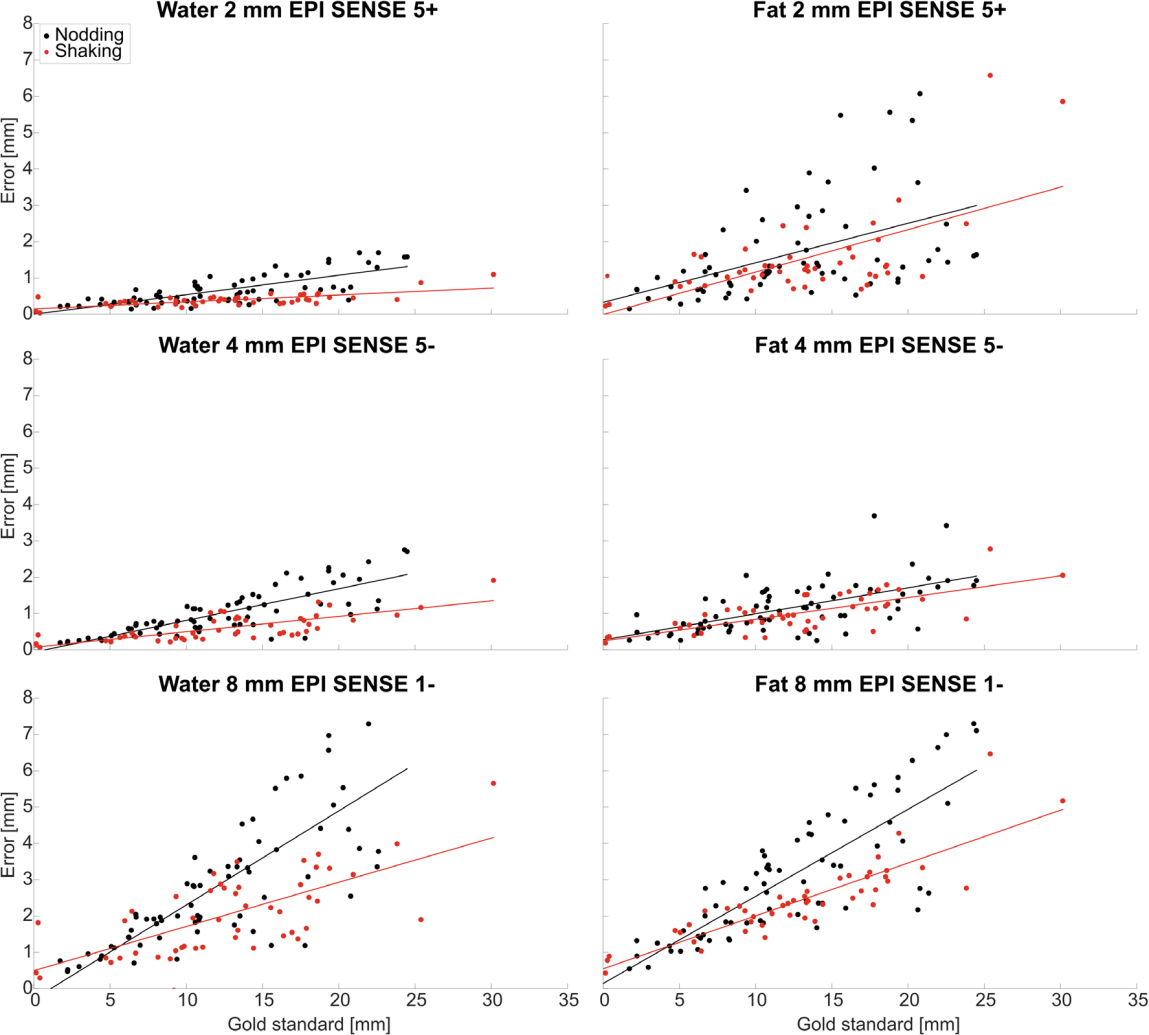
Motion score of the navigator errors plotted against the motion score of the gold‐standard realignment parameters, together with the corresponding linear fits for selected water and fat navigators. Fits for “nodding” and “shaking” experiments were estimated separately. The fit value at 10‐mm gold‐standard motion score was used as the indicator of navigator accuracy in following figures. The navigators shown here all used PF, and were cropped to 160 mm in the feet–head dimension.

**FIGURE 6:**
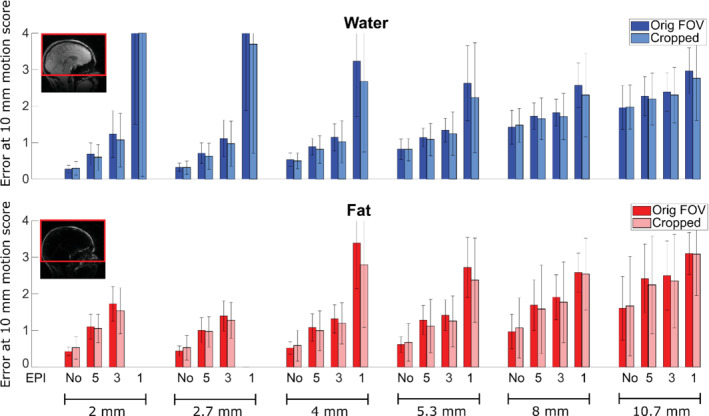
Navigator accuracy for water (blue) and fat (red) navigators with and without FOV cropping in the feet–head dimension. The main bars are the navigator error linear fit values at 10‐mm gold‐standard motion score (see Figure 5). The error bars are the fit residual standard errors, indicating goodness of fit. Shown here are the results from nodding experiments and EPI navigators with PF and negative blip directions. “EPI 5” denotes EPI‐SENSE 5, and so on. The red box in the insert image shows the FOV with cropping.

**FIGURE 7:**
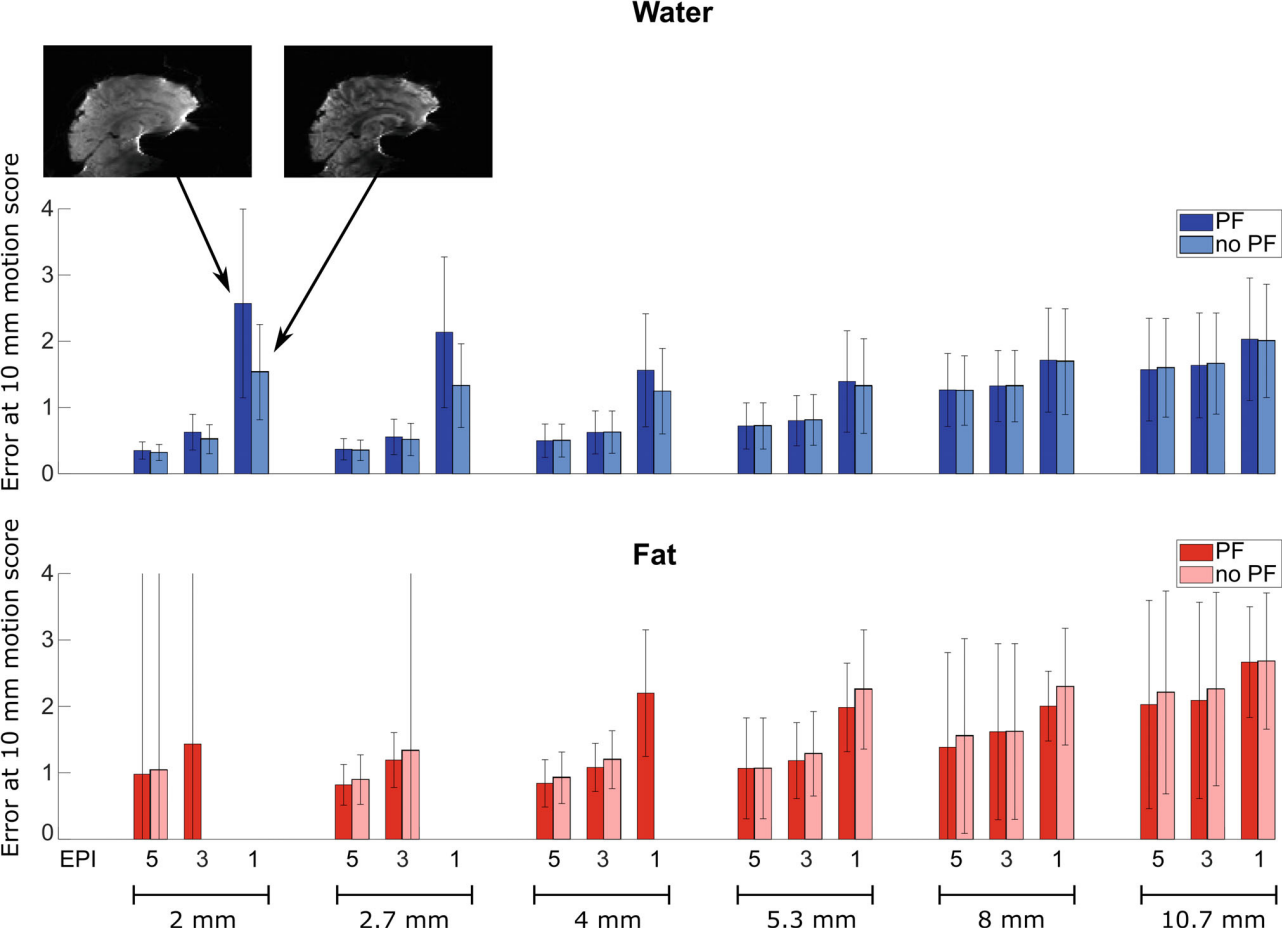
Navigator accuracy results for water (blue) and fat (red) navigators when shortening the TE by PF. Results are shown for shaking experiments and navigators with negative EPI blip direction and FOV cropping. The insert images are examples of specific navigators with/without PF. The water navigators tend to show a higher error with PF, especially for the long readouts. In contrast, the fat navigators show overall slightly smaller errors with PF, and PF allows more fat navigators to be included in the analysis (as maximum TE of 9 ms was allowed for fat navigators, due to the short T_2_* of fat).

**FIGURE 8:**
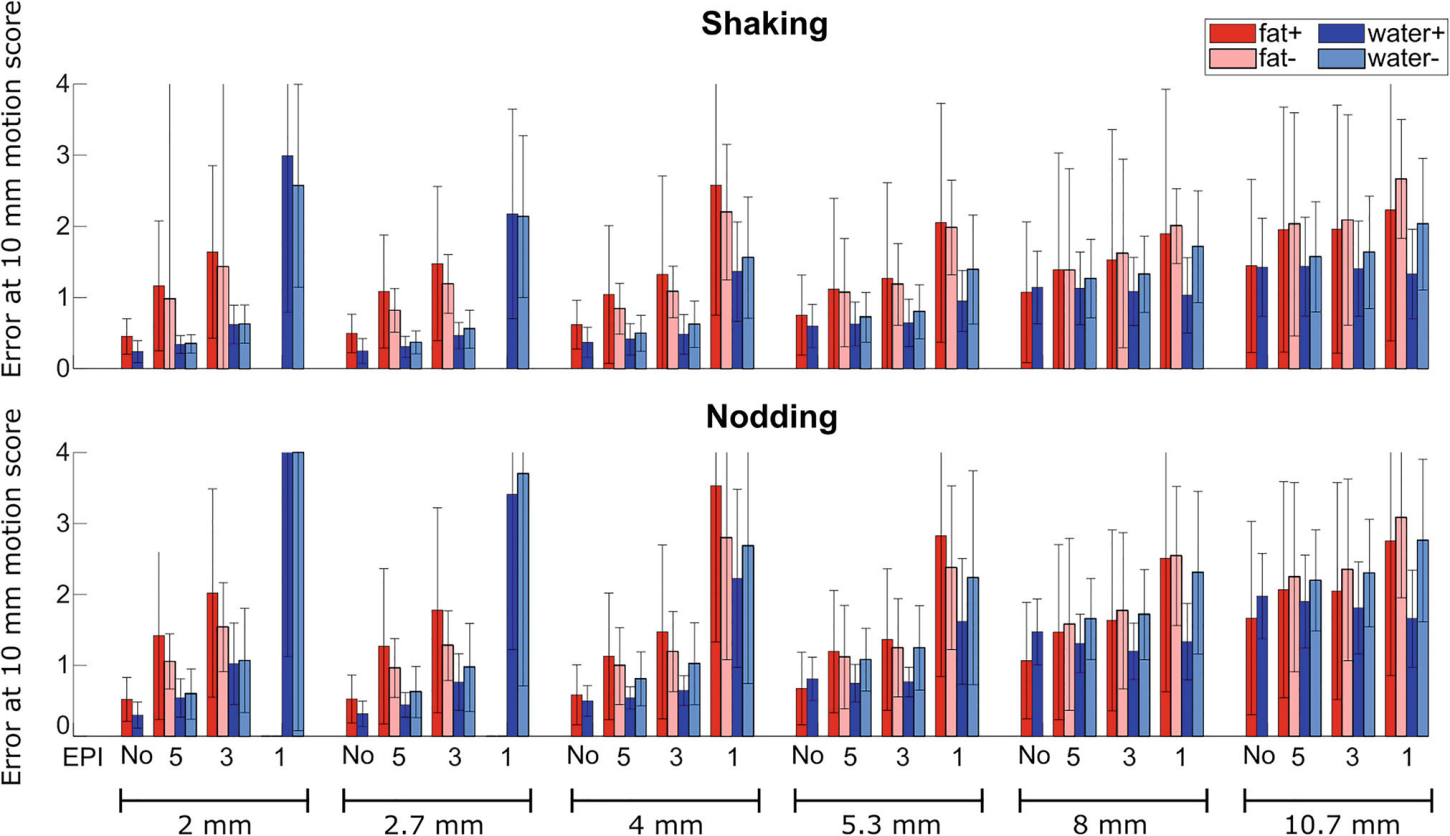
Navigator accuracy results for water and fat navigators with both positive and negative blip gradient polarities from both shaking (top) and nodding experiments (bottom). Navigators shown are with PF and cropping of the FOV. Positive blip gradient polarity results in mostly image stretching in frontal brain regions, while negative blip gradient polarity results in mostly image compression in frontal brain regions (see Figure 2).

Of the 5 × 20 × 2 volumes acquired, 10 were used as reference volumes, one was discarded due to a scanner error, one was discarded as it was a very severe outlier, and 72 were discarded as a gold‐standard rotation parameter was > 10º, resulting in 47 remaining movements for shaking motion, and 69 for nodding motion for further analysis. The realignment parameters from the gold standard and for an example navigator are shown in Figure 4, together with motion score of the gold‐standard parameters and the navigator error. The navigator error was generally observed to increase with the size of movement, as illustrated in Figure 5 for six selected navigators. The increase was typically steeper for nodding than shaking movements, especially for the water navigators, which is why linear fits were estimated separately for the data from nodding and shaking experiments. The linear fit values at 10‐mm gold‐standard motion score were used to compare accuracy among the different navigators (Figures 6, 7, 8).

Cropping the FOV in the feet–head dimension yielded a small improvement in navigator accuracy for EPI navigators in terms of the fit value at 10‐mm motion (main bars, Figure 6), although an increase in fit residuals standard error (error bars, Figure 6) was observed for some navigators, indicating less stable realignment performance. Notably, the accuracy appeared to be slightly better without cropping for the non‐EPI navigators.

Shortening the TE with partial Fourier sampling lowered the accuracy of the water navigators with the longest readout and TEs (2 mm, 2.7 mm, and 4 mm EPI 1; Figure 7), whereas partial Fourier had little effect on the accuracy of water navigators with shorter readouts. Fat navigators overall performed better with partial Fourier. Moreover, partial Fourier allowed for more fat navigators to be examined, as more scans fell within the limit of maximum TE of 9 ms for the fat navigators.

The accuracy clearly improved with higher resolution for non‐EPI navigators (Figures 6 and 8). When using EPI, there was a tradeoff between increasing resolution and increasing distortions. At low resolution, the use of EPI had a relatively small effect on the accuracy, although there was a difference between positive and negative blip gradient polarities, especially for the water navigator in which the positive blip direction gave the best accuracy (Figure 8). At medium and higher resolution, there was a larger effect of the use of EPI, favoring the shorter readout durations both for the water and fat navigators. The errors were typically higher for “nodding” than “shaking” movements. Fat navigators with EPI were typically less accurate than the equivalent water navigators (especially when considering the error bars), whereas without EPI the results were more mixed, and fat navigators without EPI and 5.3–10.7 mm voxel size showed higher accuracy than the corresponding water navigators in the “nodding” experiments.

**FIGURE 9:**
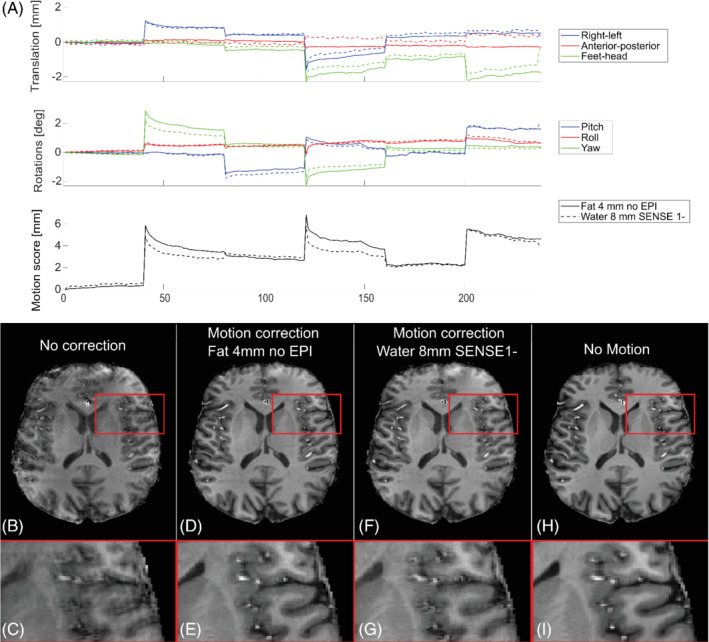
Retrospective motion correction using two different interleaved navigators. Differences in the motion traces (A) between the 4‐mm fat navigator (solid lines) and 8‐mm water EPI navigator (dashed lines) resulted in differences in image‐correction quality (D, E vs F, G). The 4‐mm fat navigator showed the best results.

Figure 8 shows that the 8‐mm EPI 1 water navigator had a much larger error (fit value at 10‐mm motion score was 1.5–2.5 mm) than the 4‐mm fat navigator without EPI (fit value at 10‐mm motion score was 0.5 mm). Navigators similar to these were used for the retrospective motion correction of the T_1_‐weighted 3D‐MPRAGE sequence and resulted in clearly different realignment parameters (Figure 9A). Both navigators yielded an improvement in image quality compared with the non–motion correction reconstruction, but a better gray‐matter/white‐matter differentiation was observed with the 4‐mm fat navigator than with the 8‐mm water EPI navigator (Figure 9E,G), confirming higher accuracy for the 4‐mm fat navigator. Some artifacts remained, especially frontally, for both the motion‐corrected reconstructions compared to a reference scan without motion (Figure 9D,F,H).

## 4 DISCUSSION

In this study we investigated the effects of different parameter choices on the accuracy of volumetric motion navigators at 7 T. We assessed water/fat selectivity, different spatial resolutions, distortion, and contrast effects of EPI with different SENSE factors, TE shortening with partial Fourier, effects of positive versus negative blip gradient orientation, and effect of cropping the FOV in the feet–head direction.

The simulation study shows some of the tradeoffs between the different acquisition parameters, and can help guide the choice for a navigator that has both the required accuracy and can still be acquired within the available time in the sequence. The time constraints on the navigator are sequence‐dependent, but also dependent on the type of motion correction, as shorter navigators are beneficial for prospective correction in which reconstructions and realignments of the navigators have to be performed before corrections can be applied in real time.

We observed navigator accuracy to decrease with increasing voxel size for non‐EPI navigators, which agrees well with a previous study by Gallichan et al.^7^ With EPI, the accuracy was clearly depending on the BW/pixel, leading to low accuracy for low BW/pixel (such as the high‐resolution SENSE 1 acquisitions), whereas lower resolution and/or higher SENSE factors led to a smaller penalty for using EPI. We observed differences in accuracy between positive and negative blip gradient polarities. For the water navigators, positive blip gradient polarities gave the best accuracy in most cases. This could be related to the fact that in the frontal regions of the brain, positive blip gradients mostly lead to image stretching, while negative blip gradients lead to image compression, and thus loss of information for the water navigators (Figure 2). This effect is not as pronounced for the fat navigators, as they are relatively sparse (the thin fat layer appears to be merely bent, and stretching or compression is not pronounced), and for the fat navigators there was not a clear preferred blip gradient polarity.

The navigator accuracy of fat EPI navigators was generally worse than for the corresponding water EPI navigators. The fat signal is only located around the brain close to air–tissue interfaces, and the portion of severely distorted voxels is therefore likely higher for fat than water EPI navigators. Furthermore, the short T_2_* of fat could play a role, as well as the fact that the fat images are sparse, meaning that large movements will lead to only few overlapping voxels between the moved fat image and the reference fat image, which we hypothesize makes the realignment algorithm less robust than for water navigators.

We found that shortening the TE with partial Fourier was beneficial for the fat navigators, which was expected as the fat signal has a rather short T_2_*. For water navigators with long readouts, we observed best accuracy without partial Fourier (long TE). An explanation could be that the most severely distorted areas have more severe signal dropout with a long TE, and are thus weighted less in the registration. Additionally, it was observed that the contrast between gray matter, white matter, and CSF improved with the long TEs, which should benefit registration. Partial Fourier reconstruction algorithms typically lead to either slight blurring or ringing, depending on the homodyne filter used,^17^ which could decrease the accuracy of navigators with partial Fourier, but that effect was not simulated in this study.

We observed that cropping the FOV in the feet–head dimension was beneficial for the EPI navigators. This is likely due to the large distortions that are present in the lower head and spine. In contrast, without EPI, especially the fat navigators showed the best accuracy without cropping. We speculate this again to be related to the fat images being rather sparse, so removing part of the FOV could make the registration less robust. Instead of cropping the FOV in the feet–head dimension, more sophisticated masking strategies might improve the results in some cases,^18^, ^19^ or an acquisition using an oblique slab to avoid more of the frontal sinuses with high susceptibility gradients.

A general trend for all types of navigators (both with/without EPI) was that larger movements led to larger navigator errors. This agrees with the results by Gallichan and Marques,^7^ where they compare navigators with different resolutions and parallel‐imaging acceleration (non‐EPI). An explanation could be that with a large movement, the starting point on the realignment cost function surface is far from the global minimum; therefore, the risk of being caught in a local minimum is higher than with a small movement.^20^ Because the coil is stationary when the head moves, the bias field from the coil sensitivities also changes with movement in the head frame of reference, and a larger movement gives more differences in terms of bias field. This could affect the realignment accuracy and is hypothesized to affect the gold standard less than the other navigators, as the parts of the head closest to the coil elements (where the bias field is strongest) are removed on the gold standard, due to the brain extraction. We chose to exclude movements with a rotation parameter > 10º from our analysis, as we observed many outlying points for these big movements, which would drive the linear fitting, and therefore dominate the analysis unreasonable much compared with their relevance. Such big rotations are rarely seen, even with highly uncooperative patients,^21^, ^22^ and are also difficult to correct with retrospective corrections, as large gaps in k‐space occur with sub‐Nyquist sampling.^23^ With prospective correction there is typically a limit to how large rotation angles can be corrected in real time, to not pose too conservative limits on the gradient strength and slew rate in the starting position. The so‐called “vNAVs” have been reported to maximally correct 8º rotations,^24^ and iMOCO^14^ has a maximum limit of 15º rotations.

We observed differences in navigator accuracy between nodding and shaking of the head, typically with highest errors for nodding movements. For EPI navigators this can be explained by the typically more severe B_0_ changes occurring with nodding compared with shaking movements, also previously documented in the literature.^25^ However, a difference between nodding and shaking was also observed for the non‐EPI navigators (although smaller than for EPI), which would have a different source of error. We speculate differences in transmit and/or receiver coil sensitivities with motion, as mentioned previously. This effect is enlarged at 7 T due to the reduced RF wavelength, and more localized receiver profiles for array coils. The effect of receiver profiles could be reduced by using the volume transmit coil in receive mode for the navigator scans, but this would remove the larger benefit of applying parallel imaging for scan‐time reduction. Alternatively, a bias field correction can be applied for every navigator volume before registration, although this will increase processing time.

In this work we only considered the most widely used sum‐of‐squared differences–based cost function for registration, but other cost functions might be better at dealing with navigator differences with motion, such as cost functions based on mutual information^26^, ^27^ typically used for intermodality registration. However, registration time is longer for such histogram‐based registrations, making them less relevant for real‐time motion correction. Despite instructing the volunteers to lie still during each Dixon volume, slight drifting movement could take place. The low‐resolution navigators are generated from the central k‐space part of the gold standard, so if subjects systematically had another position during the acquisition of the central part of k‐space compared with the periphery, the comparison between resolutions may be biased. However, considering this is a 3D scan with linear phase‐encoding order, different high‐frequency components in the image are acquired at time points separated throughout the scan. Furthermore, drifting and thereby possible artifacts are unlikely to be identical in the reference image to which it is aligned. We therefore expect the possible motion artifacts to mostly add noise‐like effects to the comparison. If there are any systematic effects, we expect them to be a slight underestimation of the navigator errors for lower‐resolution navigators, due to possible blurring of the gold standard. Of other study limitations, it should be mentioned that only the effects of parallel imaging on EPI distortions and TE were simulated, whereas the effects on SNR and other artifacts (eg, coil sensitivities not geometrically matching the navigator scan, due to distortions or motion^28^) were not simulated, which will likely degrade the accuracy compared with the results shown here. Effects of uncompensated eddy currents and gradient delays were not simulated, but if not using dynamic ghost correction^29^ it is expected that EPI ghosting levels may depend on head position. Varying ghosting levels may potentially affect registration accuracy, although we expect the effect to be small due to the typical low intensity of ghosts compared with the main image. Here we investigated the impact of EPI acceleration on navigator accuracy with parallel imaging factors up to 5. Increasing it further could be beneficial in terms of BW/pixel, but will at some point lead to severe SNR penalties and parallel‐imaging artifacts. Acceleration in the third spatial direction was not explicitly discussed in this work, as this direction does not induce distortions as long as phase encoding is used. We only considered acceleration with EPI, but there are more techniques to accelerate navigator acquisition such as spiral readouts,^19^ compressed sensing, and deep learning reconstruction. One could consider these more advanced navigator reconstructions, especially with retrospective motion correction where reconstruction times are not critical; however, care should be taken to investigate the accuracy of such navigators with motion.

An important question is what level of accuracy is required to obtain acceptable image quality. Maclaran et al^30^ analyzed how much tracking noise can be allowed on the in‐plane translation realignment parameters coming from, for example, navigators, and concluded that the standard deviation of the navigator noise should be only a fraction of the voxel size to be unnoticeable, although this depends on the SNR and the contrast in the image. In this work, we focused more on the bias in the realignment parameters when actual motion is occurring. We demonstrated that with retrospective motion correction of an MPRAGE with five discrete movements equally spread over the acquisition time, image quality was clearly better with a 4‐mm non‐EPI fat navigator than with an 8‐mm EPI water navigator. However, it is difficult to answer in a broad sense of what level of bias can be tolerated, as it will depend on the frequency and timing of motion in combination with the scan parameters of the high‐resolution host scan. For example, with large movements close to the center of k‐space, the impact on image quality will be high, and navigator accuracy will be more noticeable than with small movements in the periphery of k‐space.

## 5 CONCLUSIONS

From multi‐echo Dixon acquisitions in different positions, we were able to simulate navigators with various acquisition parameters, and compared navigator accuracy with a gold standard. We found that navigator accuracy increased with increased spatial resolution for non‐EPI scans; however, higher resolution leads to longer navigator durations. Acceleration using EPI leads to increased distortions, signal dropout and contrast effects, which reduced navigator accuracy, especially for high‐resolution navigators with little parallel imaging, leading to a tradeoff between loss of accuracy due to low BW/pixel versus loss of accuracy due to low resolution. Cropping the FOV to remove inferior regions improved the EPI navigator accuracy, whereas TE shortening with partial Fourier benefitted fat navigators, but lowered accuracy for water navigators with long readouts. Fat navigators with EPI were generally less accurate than water EPI navigators.

## CONFLICT OF INTEREST

Mads Andersen is an employee of Philips Danmark A/S. Late in the manuscript preparation phase, Malte Laustsen became an employee of TracInnovations. These employments have not influenced the design, execution, or data interpretation in the study.

## Supporting information


**Supporting Information Figure S1:** Dixon fat image (A), a simulated EPI fat navigator (B), and an acquired EPI fat navigator (C) from the same subject and with the same parameters (2 mm, BW/pixel = 55.6 Hz, TE = 11 ms, positive blip gradient polarity). For the acquired fat navigator, additional parameters were SENSE factors of 4 × 4, partial Fourier factors of 0.75 × 0.75, and flip angle of 6º
