# Supplementary material for: Accuracy investigations for volumetric head‐motion navigators with and without EPI at 7 T
Source: Magn Reson Med. 2022 May 16;88(3):1198–211. doi: 10.1002/mrm.29296 (PMC9325528; doi:10.1002/mrm.29296)
Supplement: Supplementary file 1 — Supporting Information Figure S1: Dixon fat image (A), a simulated EPI fat navigator (B), and an acquired EPI fat navigator (C) from the same subject and with the same parameters (2 mm, BW/pixel = 55.6 Hz, TE = 11 ms, positive blip gradient polarity). For the acquired fat navigator, additional parameters were SENSE factors of 4 × 4, partial Fourier factors of 0.75 × 0.75, and flip angle of 6º [file MRM-88-1198-s001.docx]

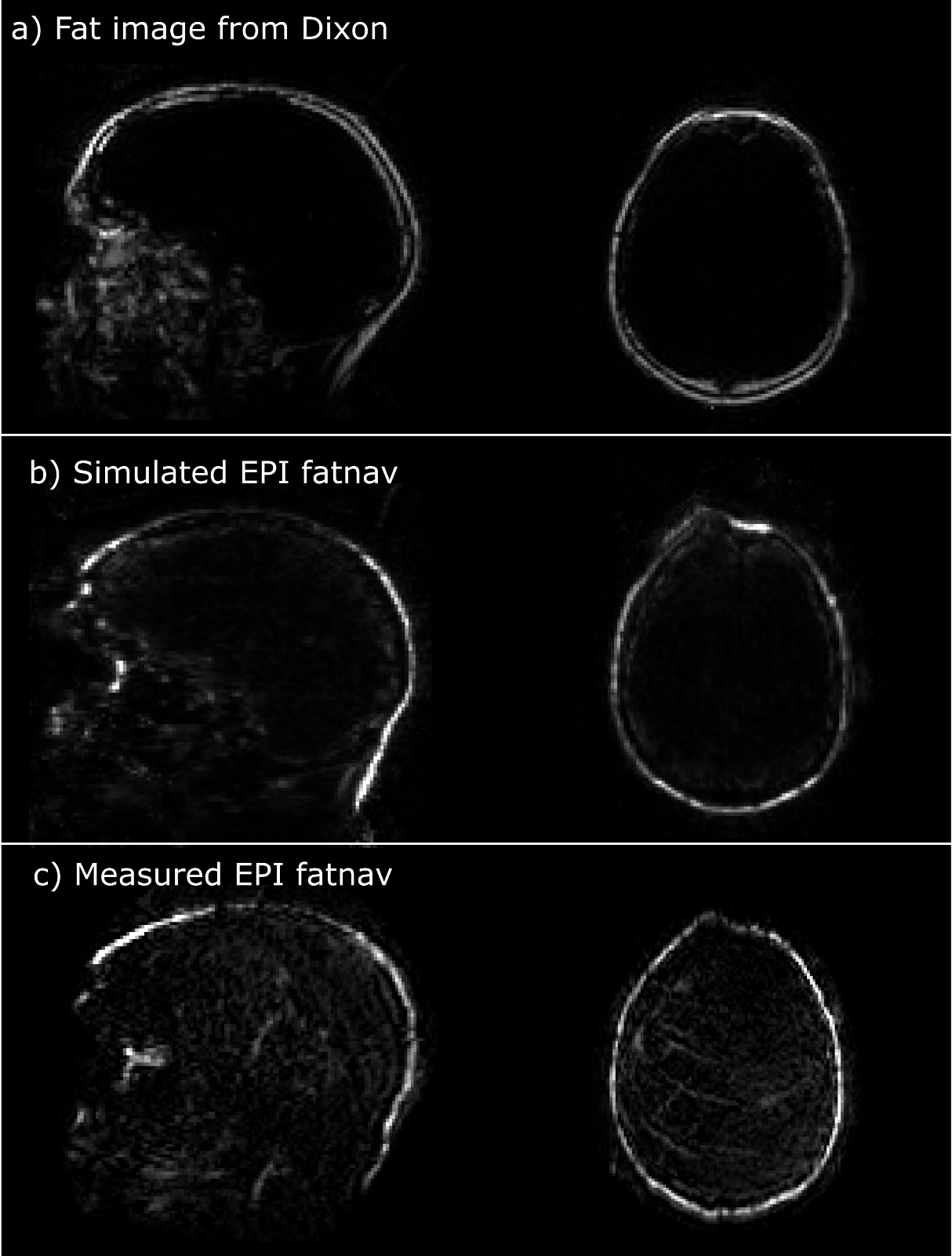


**Supporting Information Figure S1:** A Dixon fat image (a), a simulated EPI fat navigator (b) and an acquired EPI fat navigator (c) from the same subject and with the same parameters (2mm, bw/pixel= 55.6Hz, TE=11ms, positive blip gradient polarity. For the acquired fat navigator additional parameters were SENSE factors of 4x4, partial Fourier factors of 0.75x0.75, flip angle of 6°).
